# Within‐Population Genetic Structuring of the Cosmopolitan Fungus *Schizophyllum commune* in Poland and Ukraine

**DOI:** 10.1002/ece3.73840

**Published:** 2026-06-12

**Authors:** Sergiy Boiko, Yaroslav Krylov, Olena Leshcheniuk

**Affiliations:** ^1^ Institute for Evolutionary Ecology National Academy of Sciences of Ukraine Kyiv Ukraine

**Keywords:** environmental factors, Poland, *Schizophyllum commune*, simple sequence repeats of DNA, spatial differentiation, Ukraine, within‐population structuring

## Abstract

Studying the genetic features of fungal subpopulations facilitates the risk assessment associated with changes in species composition, helps develop conservation strategies, allows the evaluation of potential vectors of genotype spread, and influences environmental factors on the genetics of subpopulations. The basidiomycete wood‐decay fungus *Schizophyllum commune*, due to its widespread lifestyle and response to environmental changes, serves as a model organism for studying genetic‐evolutionary processes. In this study, we aimed to investigate the genetic structuring of the *S. commune* population in Poland and Ukraine using highly informative SSR DNA markers, to identify potential environmental factors influencing gene flow, and establish the direction of genotypes dissemination. The genetic clustering of samples within the principal components' space and creating a single network (44%) point to substantial gene flow across the overall fungal population. The Mantel test analysis of the overall dataset revealed a weak yet statistically significant correlation (*r* = 0.1415, *p* = 0.001). The Vor (4) and Olex (6) subpopulations contain the largest number of specific genotypes, as evidenced by the strong correlation between the genetic data of samples and their geographic locations. The single network of *S. commune* samples at subpopulations is formed with edge cutoff: Pol1—29%, Pol2—29%, Sha—29%, Vor—27%, Kyiv—33%, Olex—38%, Cher—32%, Kr_r—32%. The subpopulations Sha (3) and Kyiv (5) exhibit intensive gene flow. Received data strongly suggests that geographical barriers such as the Dnipro River and the Carpathian Mountains significantly affect the genetic structuring of *S. commune* subpopulations. It seems the *S. commune* genetic material is spreading from western Ukraine (Subpopulations 4, 3) to Poland (Subpopulations 1, 2), and then from the north to central Ukraine.

## Graphical abstract

In this study, we aimed to investigate the genetic structuring of the *S. commune* population in Poland and Ukraine using highly informative SSR DNA markers, to identify potential environmental factors influencing gene flow, and establish the direction of genotypes dissemination. The genetic clustering of samples points to substantial gene flow across the overall fungal population. The Mantel test analysis of the overall dataset revealed a weak yet statistically significant correlation. The data strongly suggest that geographical barriers such as the Dnipro River and the Carpathian Mountains significantly affect the genetic structuring of *S. commune* subpopulations.

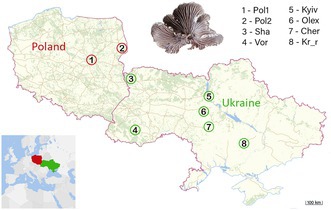

## 1 Introduction

Fungi are fundamental components of ecosystems, maintaining balance and supporting the viability of other organisms. They engage in symbiotic and parasitic interactions, contribute to environmental homeostasis, and serve as unique elements of biogeochemical cycles, particularly the circulation of carbon and nitrogen and other elements (Boiko 2021a; Clipson and Gleeson 2012; Koch et al. 2004; Singh 2021; Treseder and Allen 2000). Fungi have a global distribution, exist at considerable altitudes, depths, and under extreme climatic conditions (Inagaki et al. 2015; Li et al. 2023; Magyar et al. 2016; Mukhtar et al. 2021). Like other communities of living organisms, climate change, pollution, human activity, and other factors influence them. Habitat fragmentation and biotope destruction are affecting fungal dispersal and survival (Boddy 2016).

The population studies facilitate the risk assessment associated with changes in fungal species composition and help to develop conservation strategies. Among the vast diversity of fungi, some species are often used as model organisms or systems to address current issues in various scientific fields (Balasundaram et al. 2015; Boiko 2025a; Perez‐Nadales et al. 2014; Peris et al. 2023; van der Klei and Veenhuis 2006). This selection is primarily driven by their widespread dispersal, lifestyle, response to environmental changes, and unpretentious growth conditions. The saprotrophic basidiomycete *Schizophyllum commune* is ideally suited for population‐genetic and genetic‐evolutionary studies due to its pronounced ecological plasticity (Boiko 2021b, 2022a; Jan Vonk et al. 2019; Ohm et al. 2010; Traxler et al. 2021). Investigating genetic variation among populations is fundamental to understanding the processes of evolution and adaptation to various ecological conditions. Given the wide distribution of *S. commune* and its ability to form large populations, it is extremely challenging to assess regional changes, direction, and speed without appropriate methodological and statistical approaches. Studying genetic features at the subpopulation level will enable us to evaluate their heterogeneity, the dominance of genotypes in different regions, and likely vectors of their spread. Additionally, it is crucial to assess the influence of environmental factors on the genetics of fungal subpopulations and determine potential correlations. The specificity of the subpopulations' genetic structure may reflect both historical and contemporary processes associated with geographic and ecological barriers, climate change, and anthropogenic influences.

A critical aspect of population structure analysis is the selection of an appropriate marker system, the number of loci required, and their sensitivity. Simple sequence repeats (SSRs), or microsatellites, are co‐dominant, highly polymorphic DNA markers widely used in population genetics (Oliveira et al. 2006; Vieira et al. 2016). The microsatellite lengths range from a few to thousands of repeats. SSRs have an order of magnitude higher mutation rate, ranging from 10^−6^ to 10^−2^ events per locus per generation, than point mutations (Srivastava et al. 2019). The specific, highly polymorphic microsatellites are suitable for detecting the hybridization between closely related species, studying gene flow, and analyzing population structure. SSRs are under selection pressure in genomes, present in intergenic and noncoding regions, and a small proportion occurs within exons. Specific, highly informative SSR DNA markers were developed for the model fungus *S. commune* and show their effectiveness at both global and local levels (Boiko 2025a, 2025b).

Central‐eastern Europe, particularly Poland and Ukraine, presents a mosaic of climatic and landscape conditions that may influence the genetic structuring of *S. commune* populations. The spatial distribution of fungi is largely determined by microclimatic components such as temperature, humidity, light availability, winds, and soil conditions, which can vary significantly depending on the local landscape (Ianovici et al. 2013; Leandro‐Muñoz et al. 2017; Rupe 1987). Microclimatic conditions determine the fungal survival, reproduction, and distribution, and can either promote or limit fungal biodiversity at the population level. Unfavorable conditions may result in the elimination of genotypes from the population, thereby reducing its variability. Additionally, as the spatial distance between populations increases, gene flow may decrease, leading to spatial genetic structuring. Our study is peculiar as it involves dividing a large population into smaller locations, allowing us to identify factors affecting the genetic diversity of the fungus. Analyzing the genetic structure of different subpopulations and their interconnections will help determine the evolutionary direction of the spread of *S. commune* genotypes across Poland and Ukraine. Preliminary results on population structuring in eastern and southern Ukraine have yielded promising findings.

This study employs highly informative SSR markers to characterize the genetic structure of *S. commune* populations in Poland and Ukraine, identify key environmental drivers affecting gene flow, and infer the directionality of genotype dissemination.

## 2 Materials and Methods

### 2.1 Samples and Conditions of Cultivation

**FIGURE 1:**
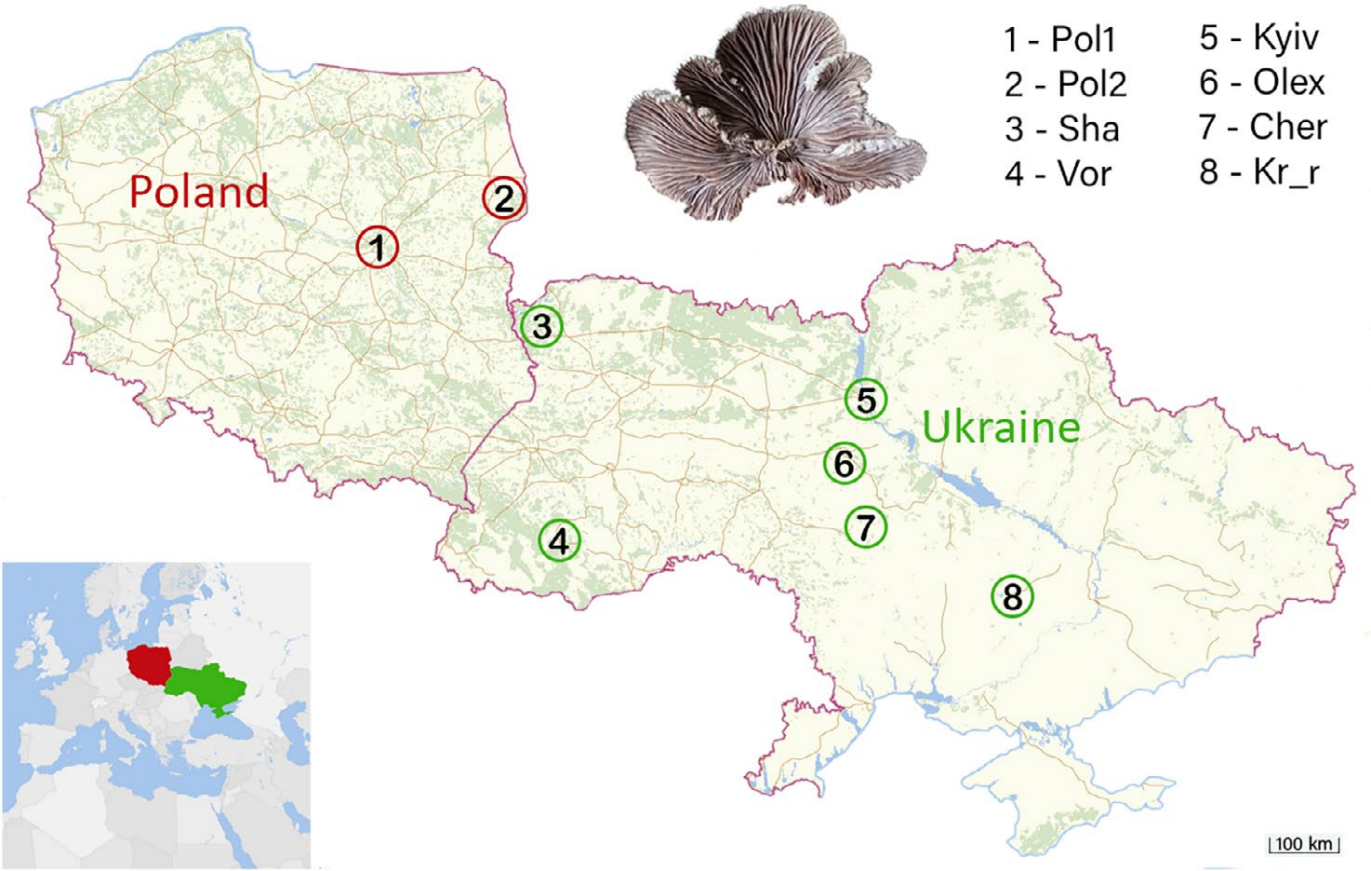
Experimental subpopulations of the *Schizophyllum commune* fungus on the territory of Poland and Ukraine. The map was created by OpenStreetMap ODbL (https://www.mapz.com).

The basidiocarp samples were selected using a simple random sampling method, ensuring that every sample had an equal chance to be chosen (Levy and Lemeshow 2008). Eight sites throughout the natural distribution of *S. commune* were the source of 169 samples. Geographic distances ranged from 75 km (Kyiv–Olex) to 1000 km (Pol1–Kr_r). The collected samples were introduced into pure culture. Pure cultures were obtained by aseptically transferring a 2–5 mg basidiocarp fragment to sterile agar, followed by repeated culturing. Cultures were stored on agar medium at a temperature of +6°С. Strains of *S. commune* are kept in the collection of the Institute for Evolutionary Ecology (Kyiv, Ukraine). The subpopulations 1 (Pol1) and 2 (Pol2) were located in eastern Poland. The subpopulations 3 (Sha), 4 (Vor), 5 (Kyiv), 6 (Olex), 7 (Cher), and 8 (Kr_r) were located in the central and western parts of Ukraine (Figure 1).

Studied subpopulations are located in forest, forest–steppe, and steppe zones with different climatic conditions. Subpopulation 4 (Vor) is located within the mountain system of central Europe—the Carpathians. Subpopulation 5 (Kyiv) partly covers the largest waterway in Ukraine, the Dnipro River. Subpopulation 6 (Olex) covers the oldest arboretum in Ukraine, Olexandria (1788).

Pure cultures were grown for 10 days at a temperature of 28°C on liquid glucose–peptone medium of the following composition (g/L): glucose—10; peptone—3; K_2_НРО_4_—0.4; MgSO_4_ × 7H_2_O—0.5; ZnSO_4_ × 7H_2_O—0.001; CaCl_2_—0.05. The nutrient medium was adjusted to pH 5 and 25 mL were poured into Erlenmeyer flasks with a capacity of 100 mL.

### 2.2 Fungal DNA Isolation

Mycelia were filtered, briefly dried, and mechanically homogenized in 2 mL tubes. Fungal DNA was extracted from approximately 50 mg of fresh mycelium. The extraction of genomic DNA was performed using a NeoPrep DNA Kit (Neogene, Ukraine) following the manufacturer's recommendations. The DNA concentration was tracked with an Analytik Jena ScanDrop2. The DNA was tested on 1% agarose gel concentrations.

### 2.3 Validation of SSR Markers

We used 34 unique primers (Table S1) specific to simple sequence repeats of genomic DNA developed for the species *S. commune* (Boiko 2022a, 2022b). Primer pairs were synthesized for special SSR DNA loci of fungus (Metabion International AG, Germany). The polymerase chain reaction was performed on thermocycler SimpliAmp (ThermoFisher, USA) using OneTaq 2× Master Mix (New England Biolabs, USA) according to the manufacturer's recommendations. PCR reactions were performed as follows: 94°C for 3 min; 35 cycles of 94°C for 30 s, 50°C–55°C for 30 s, and 68°C for 45 s; and finally, 68°C for 5 min. The size of the PCR product was confirmed using a fragment analyzer with 100 bp DNA Ladder (New England Biolabs, USA). Product formation for primer pairs was tested repeatedly. 8% polyacrylamide gel was used to analyze molecular sizing differences caused by variable microsatellite repeats.

Electrophoresis was conducted in a 0.5 TBE buffer at 10 V/cm for 60–90 min depending on fragment sizes. Gels were stained with silver following the standard protocol (Huang et al. 2018). Gel documentation was performed using the AlphaImager 2200 system (Alpha Innotech, USA). Fragment sizes were calculated in the TotalLab TL 120 software (Nonlinear Dynamics Ltd., Durham, USA) according to molecular size standards co‐separated with the SSR fragments.

### 2.4 Data Analysis

The genetic data were analyzed by using GenAlEx v6.5, PAST v5 software (Hammer et al. 2001; Peakall and Smouse 2012).

A network graph was performed to visualize and analyze the relationships between samples. Strains are represented as nodes, and the relationships between them are represented as lines (Fruchterman and Reingold 1991). We used the Dice similarity index to analyze the network of relationships between strains, as it is suitable for binary data (presence/absence).

We employed the principal components analysis (PCA) to identify hypothetical variables accounting for as much as possible of the variance in our multivariate data (Dice index). This approach allowed us to visualize effectively the genetic diversity produced by the SSR DNA markers across each genotype (Jackson 1993; Peres‐Neto et al. 2003). The method reduces the effective dimensionality of a multivariate data set by producing linear combinations of the original variables that summarize the predominant patterns in the data.

We used the Mantel test to assess the correlation between two distance matrices (Permutation: 9999). Comparing genetic divergence with geographical distances is one approach to evaluating spatial processes driving population structure (Diniz‐Filho et al. 2013; Mantel 1967). It allows us to understand spatial or environmental influences on genetic diversity. The r‐value ranges from −1 to +1. The total test evaluates overall correlation, the pairwise analysis evaluates local patterns, and the correlogram evaluates dependence on distance classes. Data standardization via Pearson and Spearman correlation coefficients preceded the analysis.

For visualizing and exploring the relationships between samples based on a similarity or dissimilarity matrix (Dice index), we used nonmetric multidimensional scaling (NMDS) (Taguchi and Oono 2005). NMDS is useful for representing complex, high‐dimensional data in a reduced number of dimensions while maintaining the rank order of distances or dissimilarities between objects. The stress value measures how well the reduced‐dimensional configuration represents the dissimilarities in the original high‐dimensional dataset. Lower stress values (below 0.2) indicate a better fit between the reduced‐dimensional solution and the original data.

Migration was estimated using RStudio 2025.05.1 and the diveRsity package (Keenan et al. 2013). Heat maps of the migration process were generated. The plots of the relative migration levels between population samples were created using the divMigrateOnline package (Sundqvist et al. 2016). The G_st_ parameter was selected to assess the relative migration between subpopulations.

## 3 Results

**FIGURE 2:**
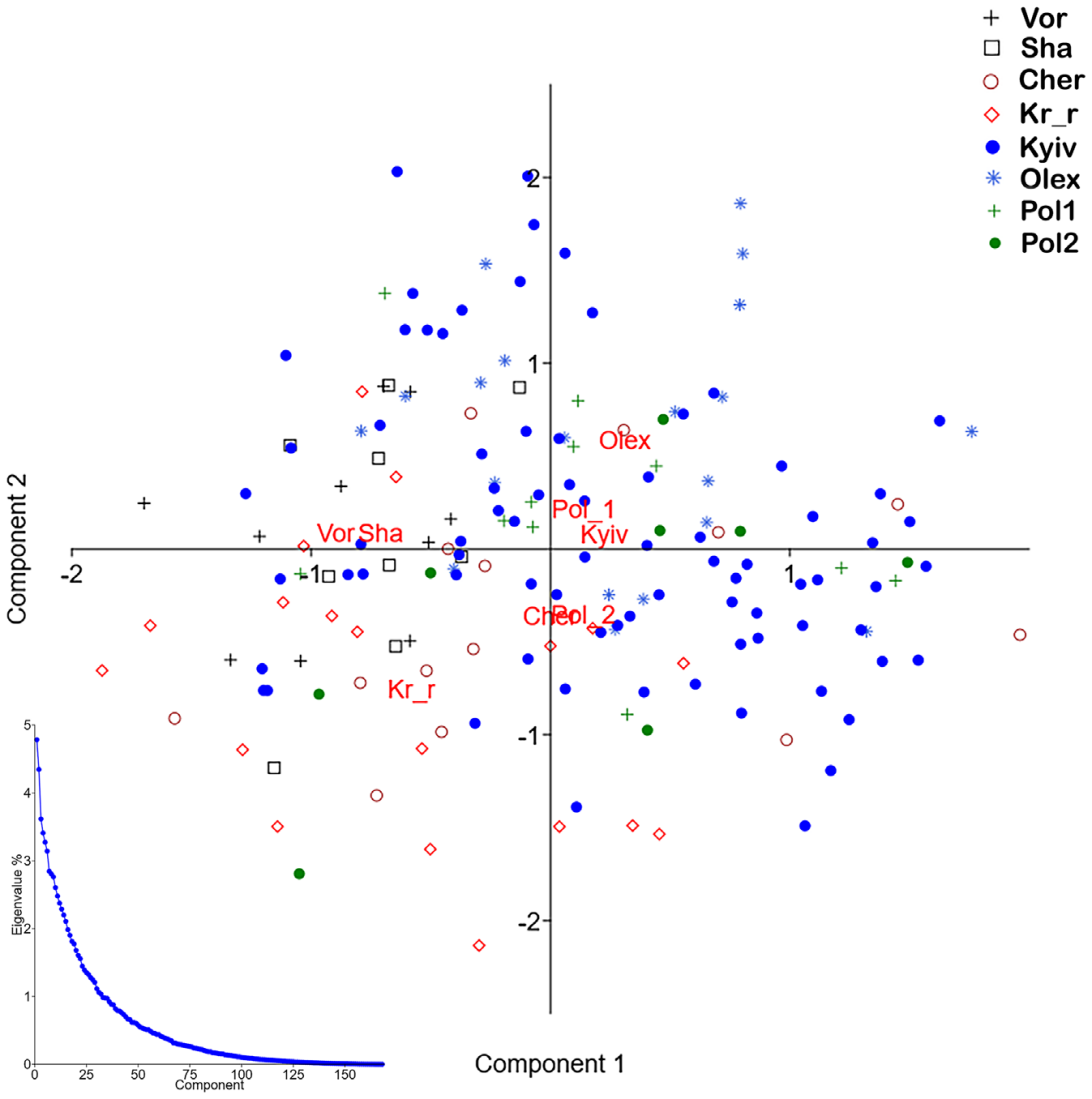
Location of *Schizophyllum commune* samples of subpopulations Vor, Sha, Cher, Kr_r, Kyiv, Olex, Pol1, and Pol2 in the space of the principal components by PCR products for 34 SSR DNA loci.

The study of the amplicon length polymorphism of *S. commune* samples allowed the determination of the position of each fungal culture in the principal components' (PC) space (Figure 2).

**FIGURE 3:**
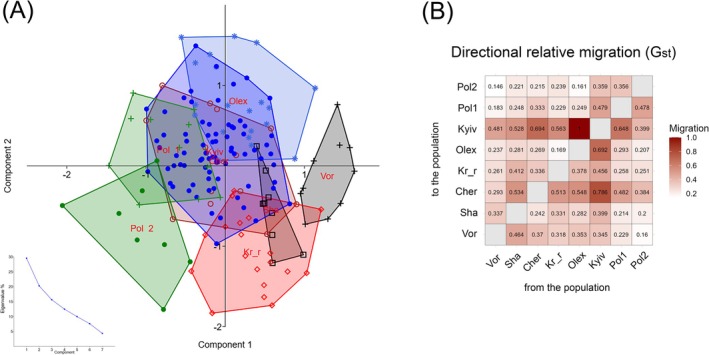
Between‐group principal component analysis of amplicons by 34 SSR DNA loci at *Schizophyllum commune* subpopulations Vor, Sha, Cher, Kr_r, Kyiv, Olex, Pol1, and Pol2 (A). Directional relative migration between fungal subpopulations (B).

The fungus's wide distribution and numerical sampling led to the formation of a single space with multitude components, where the maximum contribution is 4.78%. These values may indicate the sensitivity of the applied microsatellite marker kit. At the same time, the relatively uniform density of sample distribution in space may suggest the absence of significant barriers to gene flow between fungal subpopulations. Another pattern of *S. commune* samples is revealed when considering the samples' origin (Figure 3A). Essentially, we observed a decrease in the PC, accompanied by a significant increase in their contributions.

The first component (29.5%) separates subpopulations 1 (Pol1) and 2 (Pol2) from subpopulations 3 (Sha) and 4 (Vor). Subpopulation 5 (Kyiv) occupies a central position within the component space. The second component (20.3%) distinctly separates subpopulations 6 (Olex) and 8 (Kr_r). The third component (15.6%) enables the separation of subpopulations 4 (Vor) and 8 (Kr_r), while the fourth component (12.5%) separates subpopulations 6 (Olex) and 7 (Cher). The group PC analysis of subpopulations' distribution suggests the presence of factors that influence the direction and rate of fungus genotypes' spread. High migration flows are observed to the Kyiv and Cher subpopulations (Figure 3B). More moderate flow values are inherent in Western subpopulations, both from and into populations.

**FIGURE 4:**
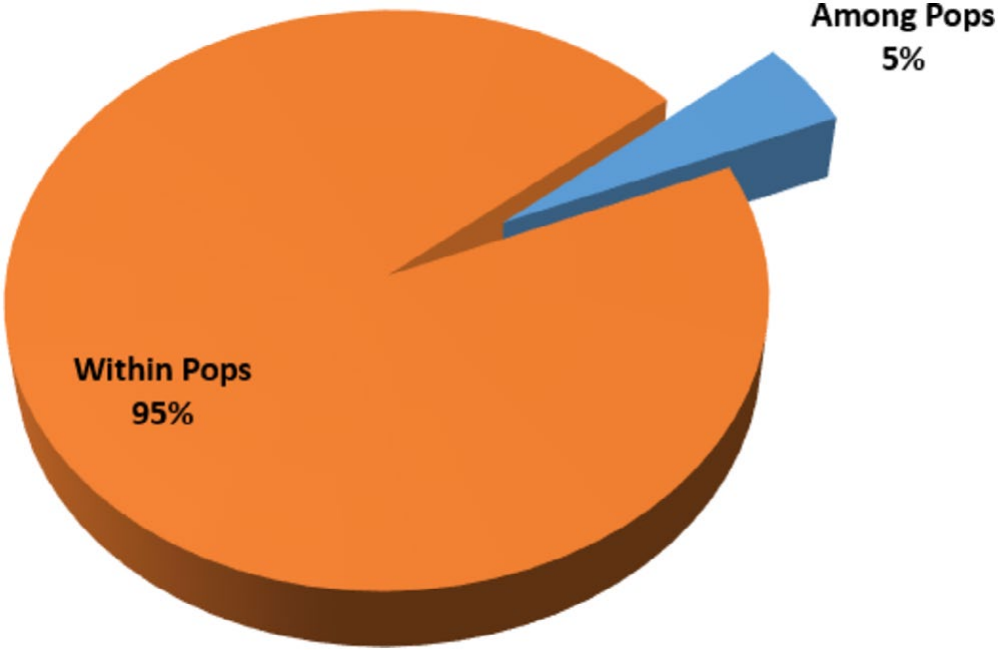
Genetic variability within/among subpopulations of the *Schizophyllum commune* fungus by AMOVA.

The relatively dense spatial distribution of fungal samples was further confirmed by molecular variance analysis (Figure 4).

According to AMOVA results, 95% of the genetic variability is within populations, while 5% is among populations. These values show significant gene flow in the total *S. commune* population, while a small percentage of subpopulation‐specific variation remains.

**FIGURE 5:**
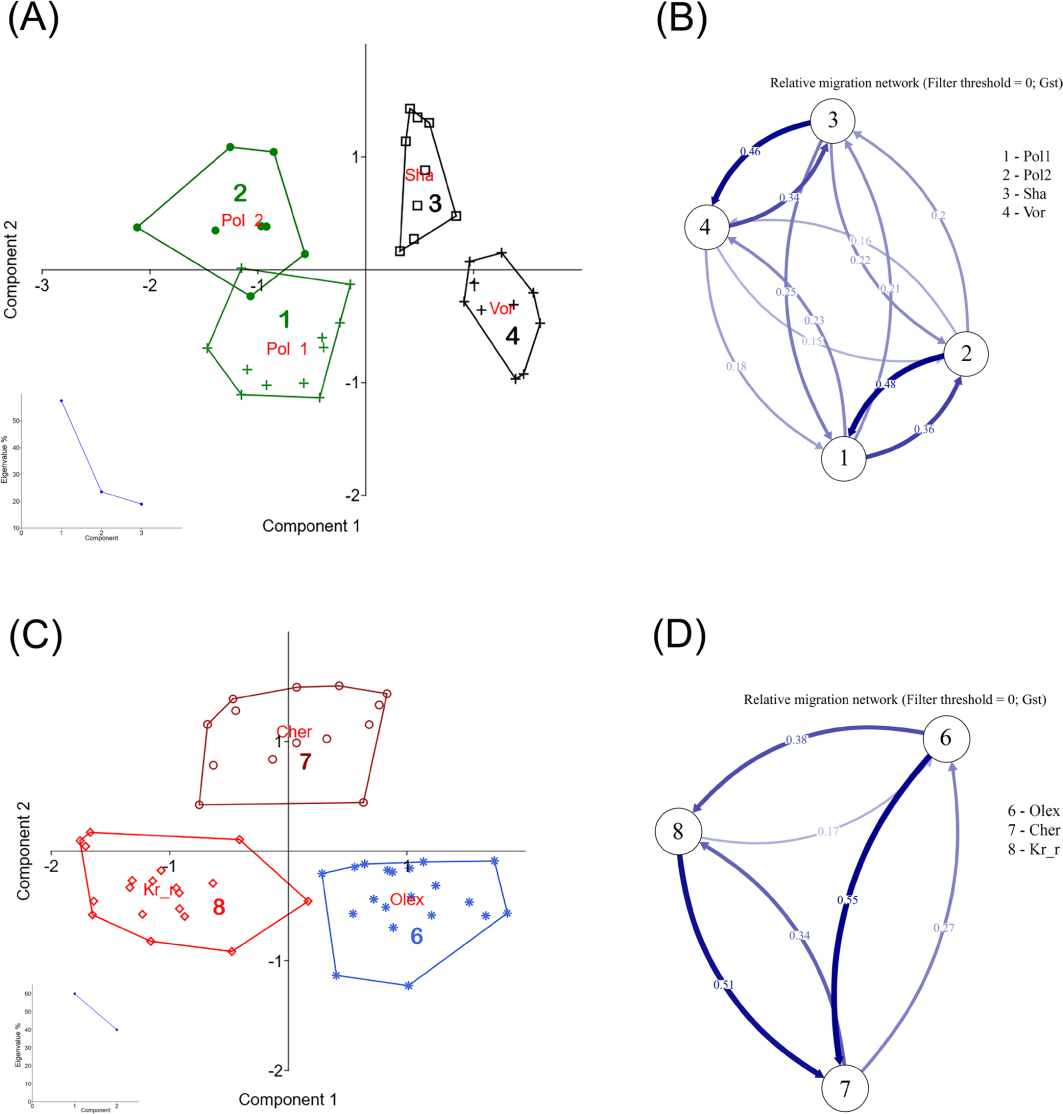
Between‐group principal component analysis and directional relative migration between fungal subpopulations Pol1, Pol2, Sha, Vor (A, B) and Olex, Cher, and Kr_r (C, D).

Our previous studies showed the high resolution of the applied set of SSR DNA markers at the local population level, which allowed us to identify specific environmental factors influencing fungal distribution in the Kyiv population (Boiko 2025a, 2025b). We applied a similar approach in this study, separating the total fungal population into discrete subpopulations to increase the sensitivity of data analysis. Primarily, our focus was on locations separated by the first principal component (PC1), namely the Pol1, Pol2, Sha, and Vor subpopulations. Considering the samples' association with certain locations, three components with high eigenvalues were identified: 57.5%, 23.5%, and 19% (Figure 5A). The fungal locations in PC space closely resemble their geographic distribution. Along the PC1, we observe a separation of Ukrainian and Polish fungal subpopulations, with the axis direction likely reflecting the historical aspect of subpopulation formation. This is further supported by the migration intensity observed among the analyzed subpopulations. The exchange of genetic material is more pronounced within the Ukrainian and Polish locations than across them (Figure 5B).

The grouping of the subpopulations Olex, Cher, and Kr_r occurs within two principal components, with contributing variances of 60% and 40%, respectively (Figure 5C). The PC1 divides two extreme subpopulations, Olex and Kr_r, while Cher occupies an intermediate position and is isolated by the second component. The intensity of gene emigration from subpopulation Olex was the highest, whereas subpopulation Cher showed the highest values of immigration (Figure 5D). In contrast, the lowest level of gene flow was detected from subpopulation Kr_r to subpopulation Olex.

**FIGURE 6:**
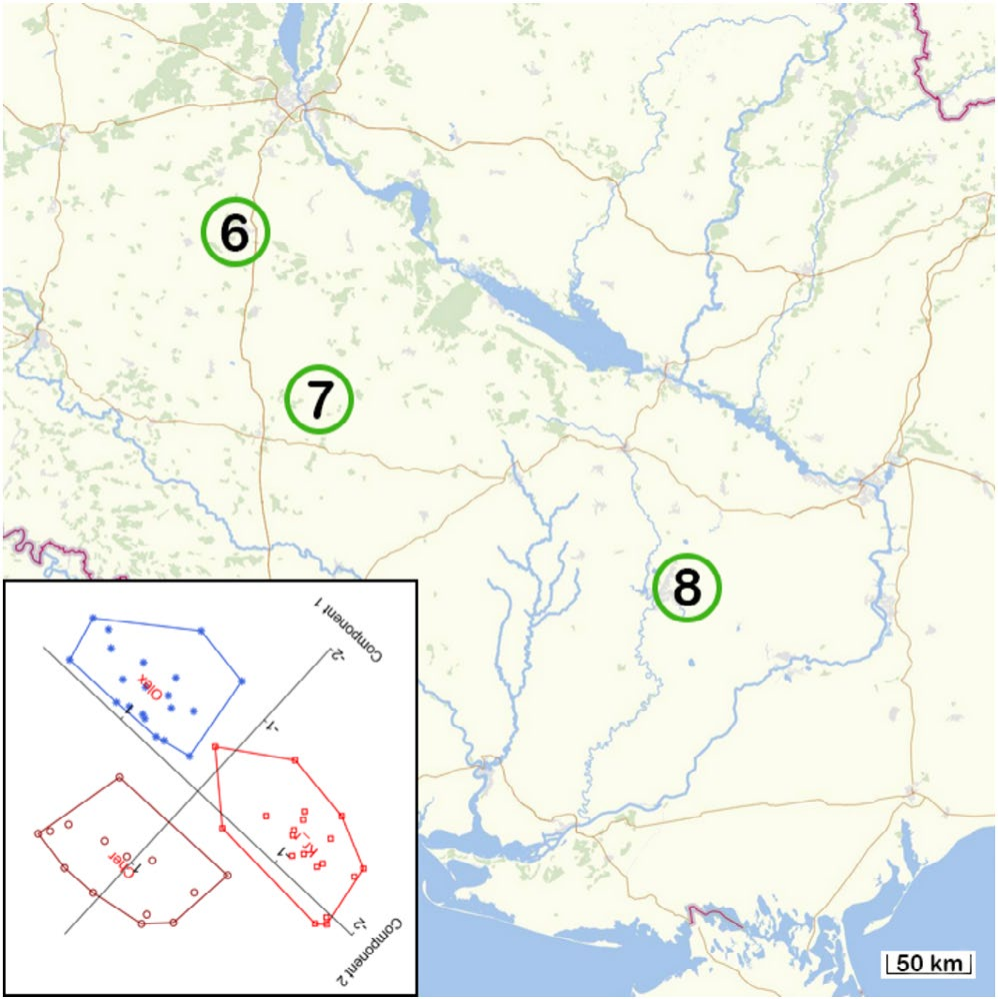
Geography location of Olex (6), Cher (7), and Kr_r (8) of the *Schizophyllum commune* fungus subpopulations, and at the principal component space.

Given that the applied set of SSR DNA markers is effective at the local scale population, we combined the geographic position of the locations with the PC coordinates (Figure 6). To compare locations, they were aligned across different systems.

As a result, the PC1 axis nearly coincides with the direction of the Dnipro River despite its considerable distance from the river (> 60 km). A similar observation was previously recorded for locations along the Dnipro River (Boiko 2025a, 2025b). A substantiated hypothesis was proposed that the Dnipro River plays a significant role in shaping the genetic profile of *S. commune* subpopulations. As we can see, the potential impact of this major waterway might be larger than we initially thought. The considerable area of location subpopulations 6–8, complex relief (Figure S1), and highly variable wind patterns (April–October) do not allow us to determine the impact of these environmental factors on the fungus's spread. Smaller systems with more controlled environmental conditions are necessary.

**FIGURE 7:**
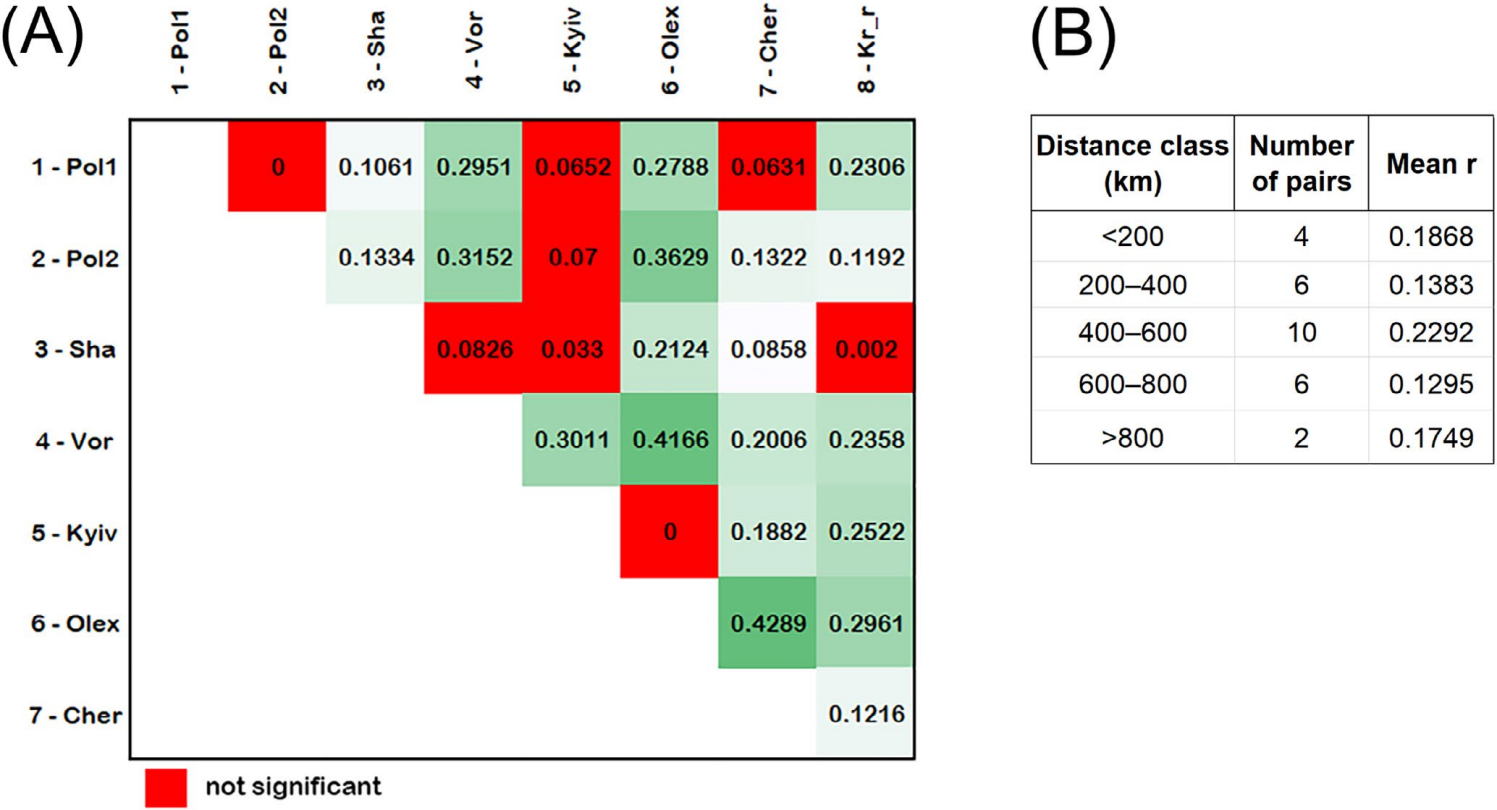
Mantel test correlation values at pairwise comparison (A) and by distance classes (B) of *Schizophyllum commune* subpopulations.

We used a Mantel test to correlate the genetic data with the spatial coordinates of fungal samples. The correlation value for the total study sample is low, but statistically significant (*r* = 0.1415; *p* = 0.001). Pairwise comparison of the correlation coefficient between all subpopulations allows for a detailed analysis of the different locations and identifies their features (Figure 7A). *R*‐values analysis revealed two subpopulations (Vor, Olex) with consistently high correlations between genetic and geographic features. This suggests the existence of a high number of specific genotypes in those populations. Notably, the Cher and Olex subpopulations exhibited the peak *r*‐value at 0.4289. The Cher subpopulation showed moderate *r*‐values in other comparisons, in contrast to Olex, indicating a dominant role of the Olex subpopulation in this pair. Subpopulations Sha and Kyiv show low or nonsignificant *r*‐values, suggesting significant gene flow within these locations.

The biological significance depends not solely on the magnitude of the correlation coefficient, but also on whether the pattern aligns with biologically plausible expectations. A weak correlation coefficient may still be biologically meaningful if it reflects underlying evolutionary processes. Pairwise grouping of locations into distance classes enables assessment of correlation significance and the detection of local, global, or barrier‐related patterns. The analysis revealed a weak global correlation between *r* and geographic distance (Pearson ≈0.19, Spearman ≈0.25), while the relationship across distance classes is non‐linear (Figure 7B). The highest mean correlation (*r* = 0.2292) was observed for the 400–600 km distance class, with a maximum value of 0.4166 recorded for the Vor–Olex pair at 435 km. Overall, the weak global correlation is attributable to nonlinearity: isolation by distance is pronounced at local and regional scales but is smoothed at larger spatial extents. These findings show biologically significant local and regional isolation, likely driven by natural barriers and landscape zonation.

**FIGURE 8:**
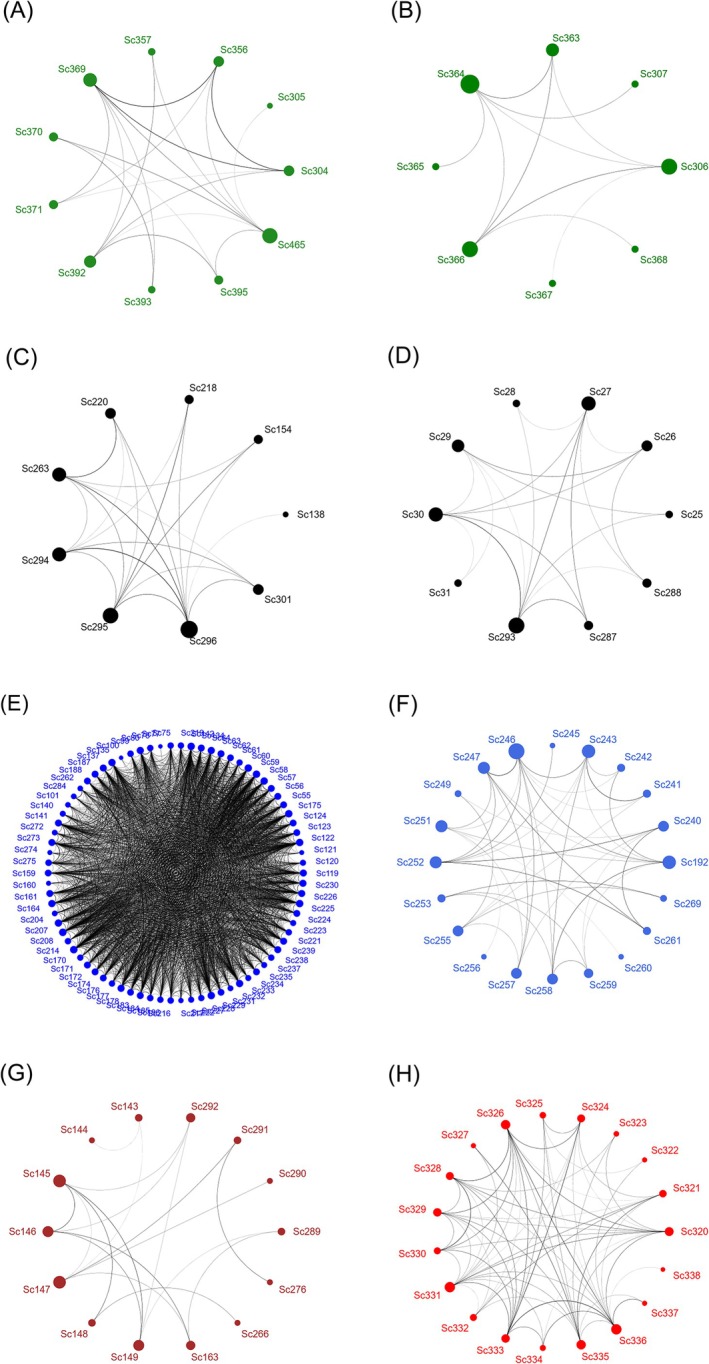
Single network formation of *Schizophyllum commune* samples at subpopulations with a cutoff value: Pol1—29% (A), Pol2—29% (B), Sha—29% (C), Vor—27% (D), Kyiv—33% (E), Olex—38% (F), Cher—32% (G), Kr_r—32% (H).

To pinpoint specific traits within *S. commune* subpopulations, we built a relationship network of samples using DNA loci for each of them. The percentage edge cutoff or similarity at which only the network formed is specific and reflects the processes occurring within the subpopulation. This value is especially significant in the comparative scheme of the study. For the Polish subpopulations, the threshold percentage for forming a unified network is identical—29% (Figure 8A,B). Combining the Pol1 and Pol2 subpopulations leads to a significant increase in percentage to 38% (Figure S2). This suggests that the new genotypes in each location had a genetic affinity with the neighboring ones. Alternatively, the Ukrainian subpopulations Sha and Vor form networks at 29% and 27%, respectively (Figure 8C,D), but their combination into a single network occurs at a much lower threshold at 32% (Figure S3). This shows that a significant influx of new genotypes from other subpopulations has occurred. In the single Sha‐Vor network, we can highlight the Sc‐28 and Sc‐31 strains, which contribute new genotype variants (Figure S3). These samples originate from the Carpathian Mountains of the Vor subpopulation.

For the largest subpopulation 5 (Kyiv), network formation is observed at a similarity of 33% (Figure 8E). Location 6 (Olex) has the highest value (38%) among all studied subpopulations (Figure 8F). This value shows high genetic homogeneity and minimal immigration processes within the location. It is important to highlight that sample Sc‐245, showing the lowest genetic relatedness to other samples within the subpopulation, was collected on the bank of the Ros River. This may suggest its “external” origin.

The formation of a single Kyiv‐Olex network occurs at a 39% edge cutoff (Figure S4), which is nearly equal to the value observed for subpopulations Pol1–Pol2. It's crucial to note that the genotype of each sample strongly influenced the threshold percentage more than sample size. The values 32% seen for the Cher and Kr_r subpopulations confirm this (Figure 8G,H). The density of the link between fungal samples within the Kr_r location is considerably higher. The reduced genetic relatedness of sample Sc‐338 shows its “external” origin. Excluding this sample from the analysis explodes the similarity value to 37%. Notably, the single network for the Cher‐Kr_r subpopulations emerges at the 36% cutoff, where sample Sc‐338 is again the limiting factor (Figure S5). It can be inferred that sample Sc‐338 does not originate from the Cher subpopulation. Most likely, to determine its origin, it is necessary to explore more eastern and southern subpopulations of *S. commune* .

Thus, among the studied subpopulations of the fungus, the most homogeneous was Olex, and heterogeneous—Vor. At a 44% cutoff, the eight *S. commune* subpopulations' sample forms a single network. The least genetically related samples were found in the Kyiv (Sc‐77, Sc‐160) and Vor (Sc‐31) subpopulations. In nature, these samples were placed in peripheral positions, respectively subpopulations, suggesting their “alien” origin.

**FIGURE 9:**
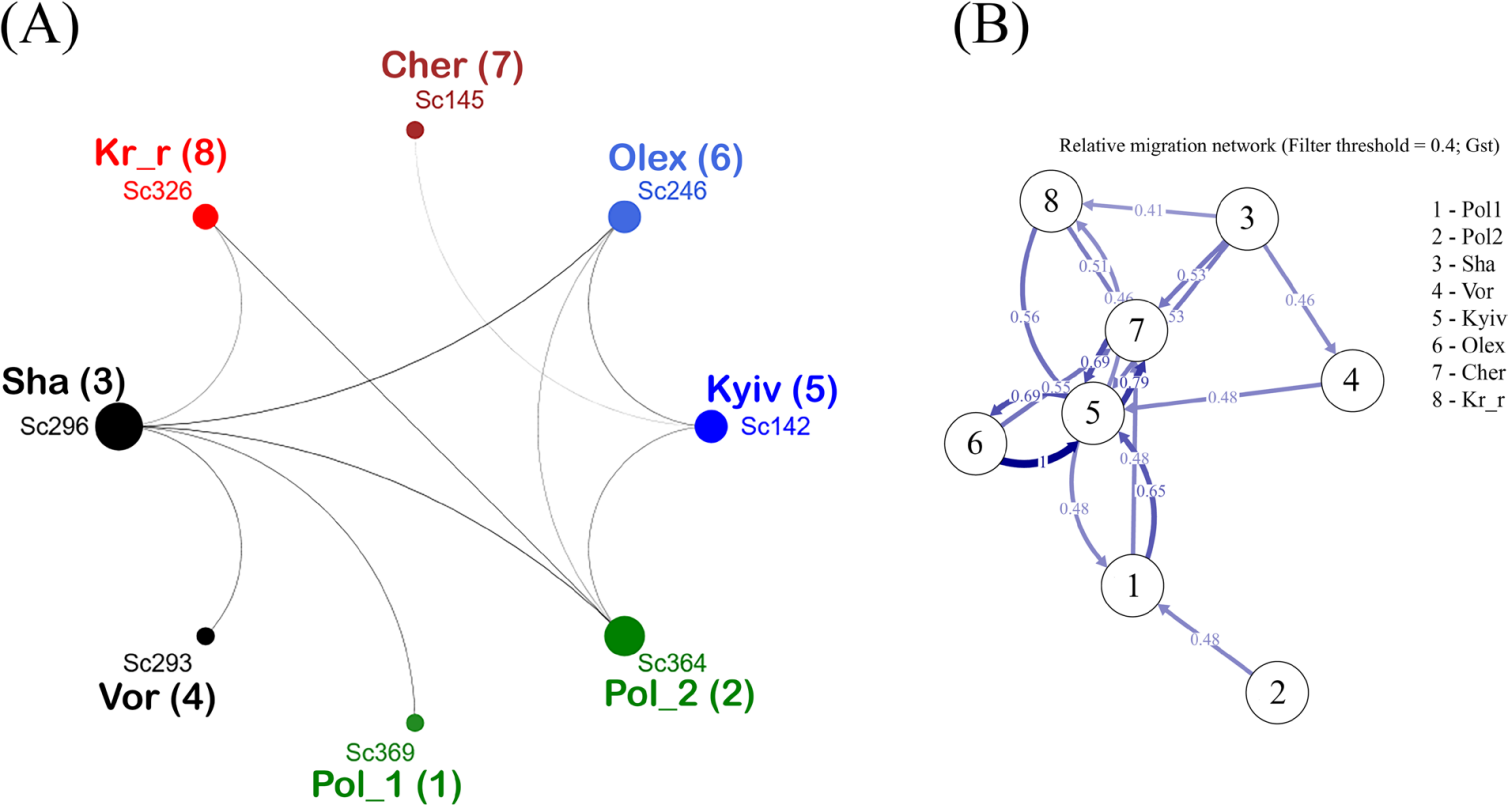
Network of eight CGA *Schizophyllum commune* samples in Poland and Ukraine at a 33% cutoff (A), and the relative migration network between eight fungal subpopulations (B).

We established evolutionary relationships between subpopulations using samples that are centers of genetic alteration (CGA) in each subpopulation (Boiko 2025a, 2025b). Constructing genetic links between CGA samples allows us to eliminate “noise” as new genotype variations and enables the determination of the origins direction of each location. The identified CGA samples for their respective locations are as follows (Figure S6): Sc‐369 (Pol1), Sc‐364 (Pol2), Sc‐296 (Sha), Sc‐293 (Vor), Sc‐142 (Kyiv), Sc‐246 (Olex), Sc‐145 (Cher), and Sc‐326 (Kr_r). The single network of samples was formed at a 33% cutoff (Figure 9A).

This value is close to the average cutoff across all locations (31%). The subpopulations Sha and Pol2 formed a more extensive network (sample associated with respective location). These represent the westernmost location in Ukraine and the easternmost in Poland, respectively. The formation of the overall migration flows among the studied subpopulations using a filter threshold confirms the substantial contribution of the Sha subpopulation to the genetic profile of the other locations (Figure 9B). From an evolutionary perspective, the formation of genetic affinity between subpopulations requires a considerable amount of time. Analyzing the genetic profile shifts and interconnections within each *S. commune* subpopulation allows us to infer the probable direction of gene flow. We applied the NMDS to assess the effectiveness of the proposed approach among *S. commune* subpopulations (Figure S7). As a result, the stress value was decreased to 0.153, which confirms the reliability of the proposed model.

## 4 Discussion

The distribution of fungi is determined by terrain, meteorological factors, the ability of spores to survive in environmental conditions, plants and animals' presence, and human activities (Aylor 1999; Brown and Hovmøller 2002; Grinn‐Gofroń and Bosiacka 2015; Kildesø et al. 2003; Magyar et al. 2016). Studying fungal dispersion involves using various techniques: spore capture using traps, morphometric analysis, genetic comparisons of different populations, and statistical modeling and simulation of fungal spread (Boutry et al. 2023; Castaño et al. 2017; Dam 2013; Garin et al. 2014). The most prevalent approach is comprehensive, combining various methodologies and considering the fungal lifestyle (Barberán et al. 2015; Geml et al. 2008; Grantham et al. 2015). The ability of fungal conidia, basidiospores, ascospores, etc. to disperse over large distances is highly dependent on conditions. Discussions about trends of fungal dispersal in nature remain relevant because no two populations are identical; each is unique. Focusing solely on spore dispersion makes it difficult to empirically measure spore spread. It is incorrect to assume that spores escaping beyond experimental setups are statistically insignificant; moreover, the viability of spores is often ignored, which is an erroneous approach. It is essential to consider as many significant factors influencing spore dispersal as possible. One of the most influential factors in fungal dispersion is water. The aquatic environment serves as an ideal medium for fungal dispersal, given that spore viability is often constrained by desiccation and abrupt temperature fluctuations (Paz et al. 2021). Water temperature fluctuations occur much more gradually than in air, and water also acts as an effective filter against UV radiation. Waterways of various scales and rates function as carriers of fungi and their spores (Madden 1997; Pang et al. 2013). Seas and lakes provide extensive surfaces over which spores and fungal fragments can move by micro‐currents and wind, whereas rivers facilitate the continuous transport of genetic material along the direction of the current (Tadych et al. 2007). The shape of fungal spores often contributes to their dispersal and adhesion to different substrates within the aquatic environment. The model basidiomycete fungus *Schizophyllum commune* possesses various adaptations and mechanisms that enhance its dispersal through water (van Wetter et al. 2000; Wessels et al. 1991; Wösten et al. 1999). Our study reveals the Ros River's flow as the reason behind the new genotype (Sc‐245) appearing in the most homogenous Olex (6) subpopulation. The spreads of the fungal genetic material at subpopulations were as follows: Olex (6), Cher (7), and Kr_r (8) (Figure 5B). Statistical data provide a reasonable basis to assert that Ukraine's largest waterway, the Dnipro River, directly influences the formation of subpopulation genetic profiles (Figure 6). Our previous research on the subpopulation Kyiv (5) confirms the critical role waterways play in the migration of *S. commune* fungus (Boiko 2025a, 2025b). Other authors have also reported similar dispersal ways for other fungal groups (Chaudhary et al. 2022; LeBrun et al. 2018; Magyar et al. 2016).

Another active mode of fungal dispersal occurs through the air via billions of spores (Dijksterhuis and Samson 2007; Golan and Pringle 2017). The spores can ascend to impressive heights thanks to wind currents and updrafts created by rising hot air, often triggered by forest fires. Fires can rapidly heat the air and generate high‐velocity updrafts (Paugam et al. 2016). Fire‐borne updrafts may promote spore dispersal in mountainous terrain (Mims and Mims 2004). Wind patterns are used to infer the atmospheric dispersal of fungi in/from different hemispheres (Muñoz et al. 2004; Schmale and Ross 2015). The atmospheric pathway fungal dispersal may involve wind and a combination of meteorological phenomena (cloud, precipitation) (Jones and Harrison 2004). In our study, subpopulation Vor (4) is located within the Carpathian Mountain system (area of 24,000 km^2^) in western Ukraine. Frequent windstorms contribute to episodic active fungal spore dispersal. Uncertain storm wind patterns in the Ukrainian Carpathians complicate forecasting potential fungal gene flow paths (Lavnyy 2017). This uncertainty can only be resolved by combining genetic methods with modern statistical approaches. Our study using microsatellite DNA showed the highest genetic diversity in subpopulation Vor (4), suggesting a large gene pool of *S. commune* in the Ukrainian Carpathians and a likely high rate of gene flow. Notably, sample Sc‐31 (Vor) showed the lowest genetic relatedness across the entire dataset.

The spatial arrangement of subpopulations 1, 2, 3, and 4 within the principal component space (Figure 5A) corresponds to their distribution under natural conditions. Comparing two coordinate systems (Figure S8), we observe a pattern similar to that in Figure 6. The axis of the 1 PC is parallel with the Carpathian Mountain system (similar to the Dnipro River). This leads us to talk about the Carpathians' impact on the genetic structure formation of fungal populations, including *S. commune* (Boiko 2026). Studies of *Armillaria cepistipes* in the Ukrainian Carpathians, supporting our assumption, showed minimal genetic differentiation between the two populations studied (Tsykun et al. 2017). Furthermore, it has been confirmed that SSR DNA markers are the most suitable for analyzing the genetic structure of local fungal populations on a small spatial scale (~50 km).

## 5 Conclusions

A comprehensive study of *S. commune* subpopulations across Ukraine and Poland has revealed several key features. The genetic clustering of samples within the principal components' space and the formation of a single network (44%) points to substantial gene flow across the overall fungal population. AMOVA data also supported this, which shows that genetic variability among subpopulations contributed to 5% of the overall variation. Subpopulations Vor (4) and Olex (6) are the most isolated among the examined, evidenced by the strong correlation between the genetic data of samples and their geographic locations. The subpopulations Sha (3) and Kyiv (5) exhibit an intensive gene flow. The single network formation within each *S. commune* subpopulation and across the overall allowed us to identify “immigrant” samples Sc‐77, Sc‐160 (Kyiv), and Sc‐31 (Vor), which are positioned at the peripheries of their respective subpopulations. Received data strongly suggests that geographical barriers such as the Dnipro River and the Carpathian Mountains significantly affect the genetic structuring of *S. commune* subpopulations. Identifying samples that serve as centers of genetic alteration has enabled us to determine evolutionary connections between fungal subpopulations. The *S. commune* fungus likely spreads from western Ukraine (subpopulations 4, 3) to Poland (subpopulations 1, 2) and then from the north to central Ukraine.

## Author Contributions

**Sergiy Boiko:** conceptualization (equal), data curation (equal), formal analysis (equal), investigation (equal), methodology (equal), project administration (equal), software (equal), supervision (equal), validation (equal), visualization (equal), writing – original draft (equal), writing – review and editing (equal). **Yaroslav Krylov:** validation (equal), writing – original draft (equal), writing – review and editing (equal). **Olena Leshcheniuk:** methodology (equal), writing – original draft (equal), writing – review and editing (equal).

## Funding

The authors have nothing to report.

## Ethics Statement

The authors have nothing to report.

## Consent

The authors have nothing to report.

## Conflicts of Interest

The authors declare no conflicts of interest.

## Supporting information


**Figure S1:** Topographic map of subpopulations 6 (Olex), 7 (Cher), and 8 (Kr_r) location.
**Figure S2:** The single network formation of *Schizophyllum commune* samples of subpopulations Pol1 and Pol2 at a 38% edge cutoff.
**Figure S3:** The single network formation of *Schizophyllum commune* samples of subpopulations Sha and Vor at a 32% edge cutoff.
**Figure S4:** The single network formation of *Schizophyllum commune* samples of subpopulations Kyiv and Olex at a 39% edge cutoff.
**Figure S5:** The single network formation of *Schizophyllum commune* samples of subpopulations Cher and Kr_r at a 36% edge cutoff.
**Figure S6:** The *S. commune* samples' network of eight locations in Poland and Ukraine at 34% (a), 43% (b), 44% (c), 47% (d) edge cutoff.
**Figure S7:** Non‐metric multidimensional scaling (Dice similarity index) of the center of genetic alteration samples of *S. commune* (stress: 0.153).
**Figure S8:** The geographic and principal component space location of subpopulations Pol1 (1), Pol2 (2), Sha (3), and Vor (4) of the *S. commune* fungus.

**Table S1:** Sequences of PCR primers for the unique SSR loci of *Schizophyllum commune*.

## Data Availability

All data generated or analyzed during this study are included in this published article or in Supporting Information files.
