# Supplementary material for: Within‐Population Genetic Structuring of the Cosmopolitan Fungus Schizophyllum commune in Poland and Ukraine
Source: Ecol Evol. 2026 Jun 12;16(6):e73840. doi: 10.1002/ece3.73840 (PMC13261372; doi:10.1002/ece3.73840)
Supplement: Supplementary file 1 — Figure S1: Topographic map of subpopulations 6 (Olex), 7 (Cher), and 8 (Kr_r) location. Figure S2: The single network formation of Schizophyllum commune samples of subpopulations Pol1 and Pol2 at a 38% edge cutoff. Figure S3: The single network formation of Schizophyllum commune samples of subpopulations Sha and Vor at a 32% edge cutoff. Figure S4: The single network formation of Schizophyllum commune samples of subpopulations Kyiv and Olex at a 39% edge cutoff. Figure S5: The single network formation of Schizophyllum commune samples of subpopulations Cher and Kr_r at a 36% edge cutoff. Figure S6: The S. commune samples' network of eight locations in Poland and Ukraine at 34% (a), 43% (b), 44% (c), 47% (d) edge cutoff. Figure S7: Non‐metric multidimensional scaling (Dice similarity index) of the center of genetic alteration samples of S. commune (stress: 0.153). Figure S8: The geographic and principal component space location of subpopulations Pol1 (1), Pol2 (2), Sha (3), and Vor (4) of the S. commune fungus. [file ECE3-16-e73840-s002.pdf]

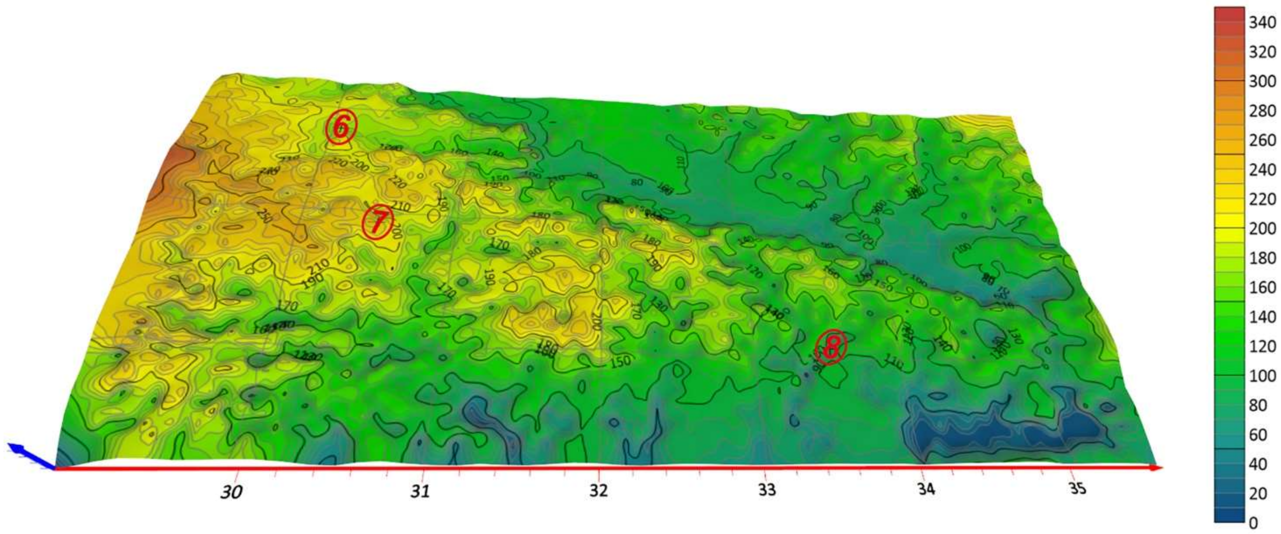

Figure S1 Topographic map of subpopulations 6 (Olex), 7 (Cher), and 8 (Kr_r) location

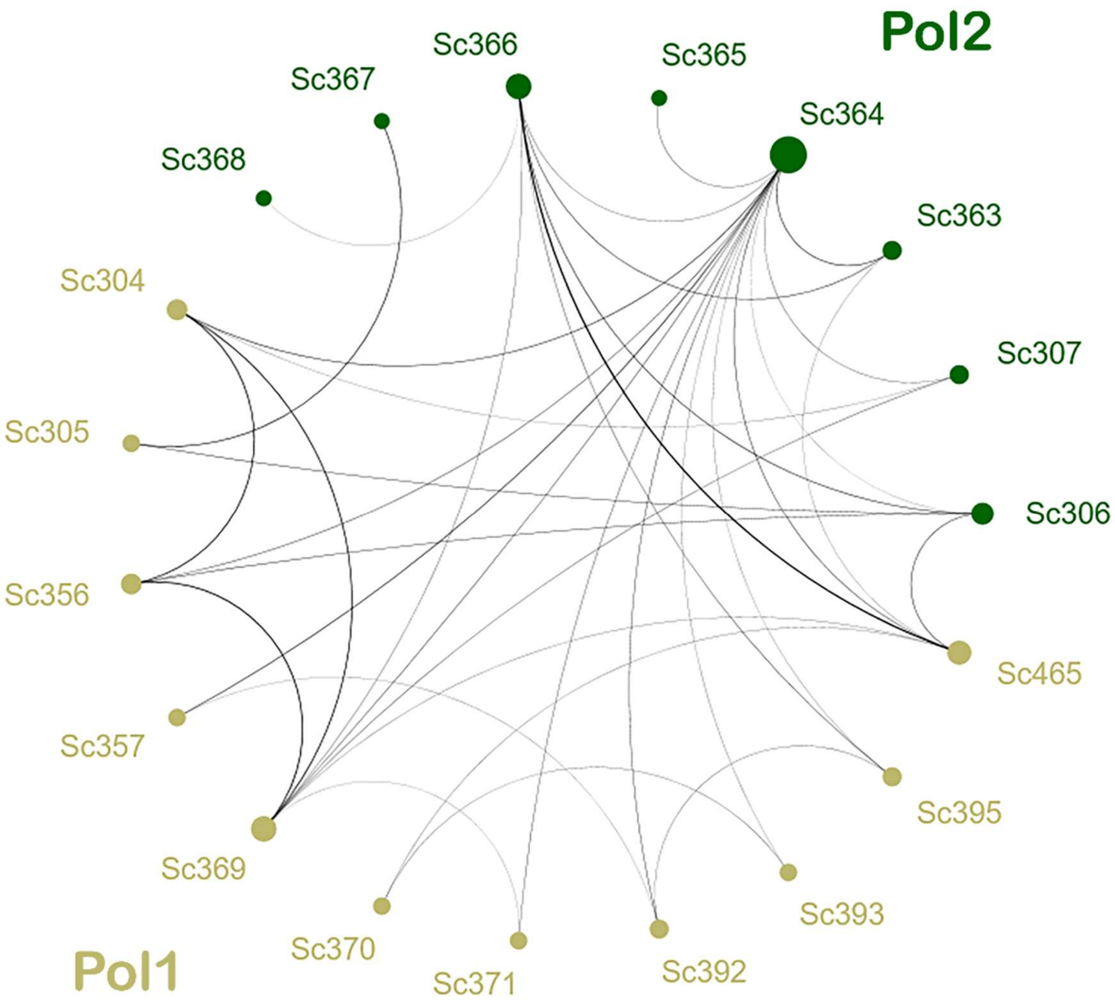

Figure S2 The single network formation of *Schizophyllum commune* samples of subpopulations Pol1 and Pol2 at a 38% edge cutoff.

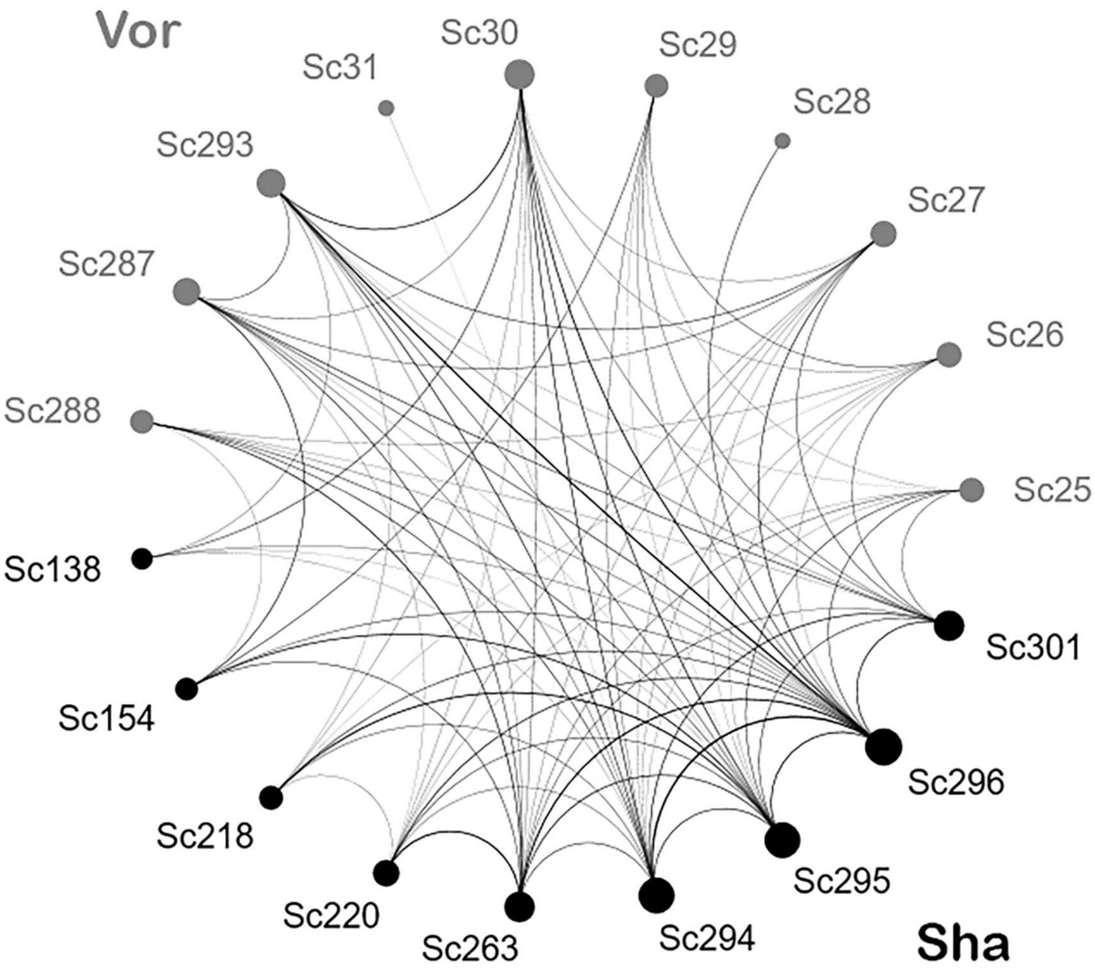

Figure S3 The single network formation of *Schizophyllum commune* samples of subpopulations Sha and Vor at a 32% edge cutoff

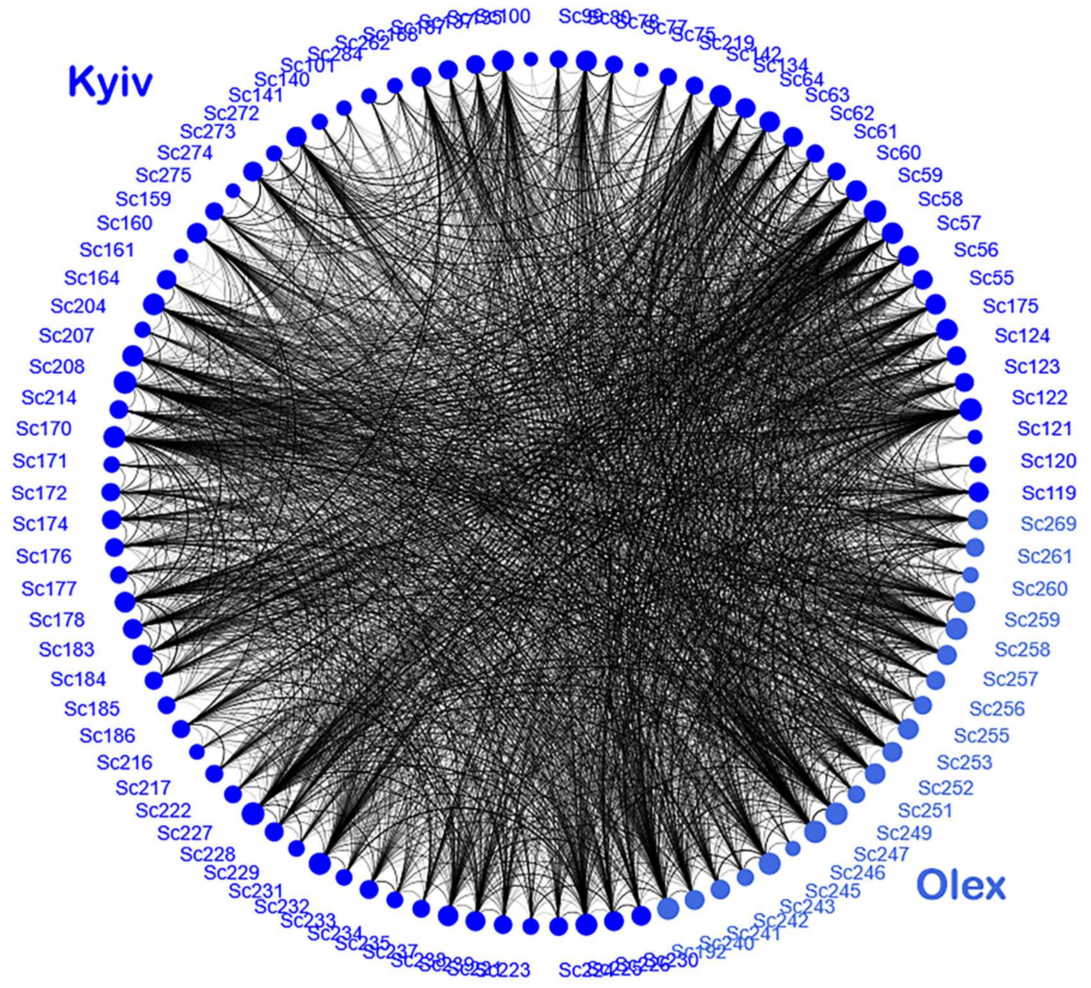

Figure S4 The single network formation of *Schizophyllum commune* samples of subpopulations Kyiv and Olex at a 39% edge cutoff

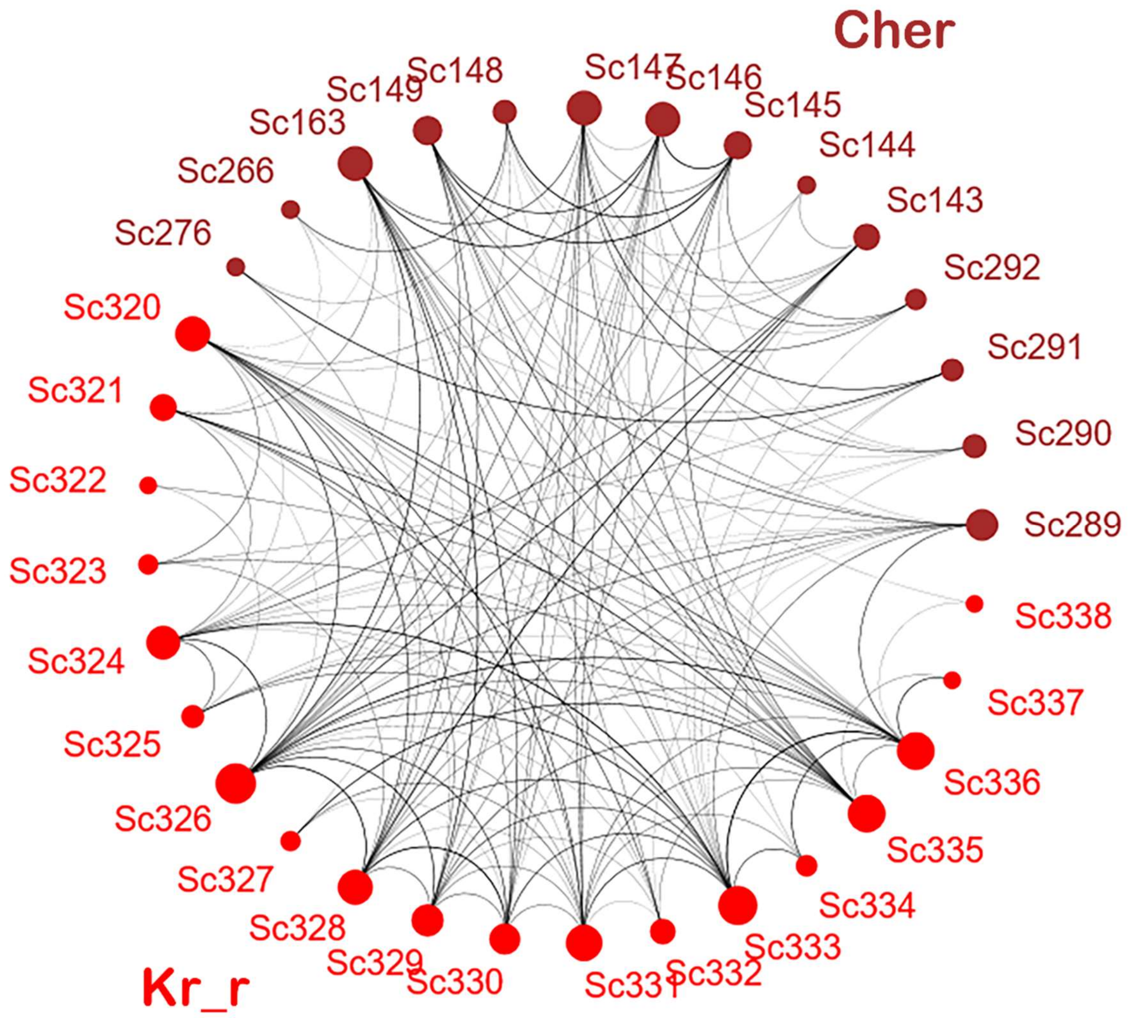

Figure S5 The single network formation of *Schizophyllum commune* samples of subpopulations Cher and Kr_r at a 36% edge cutoff

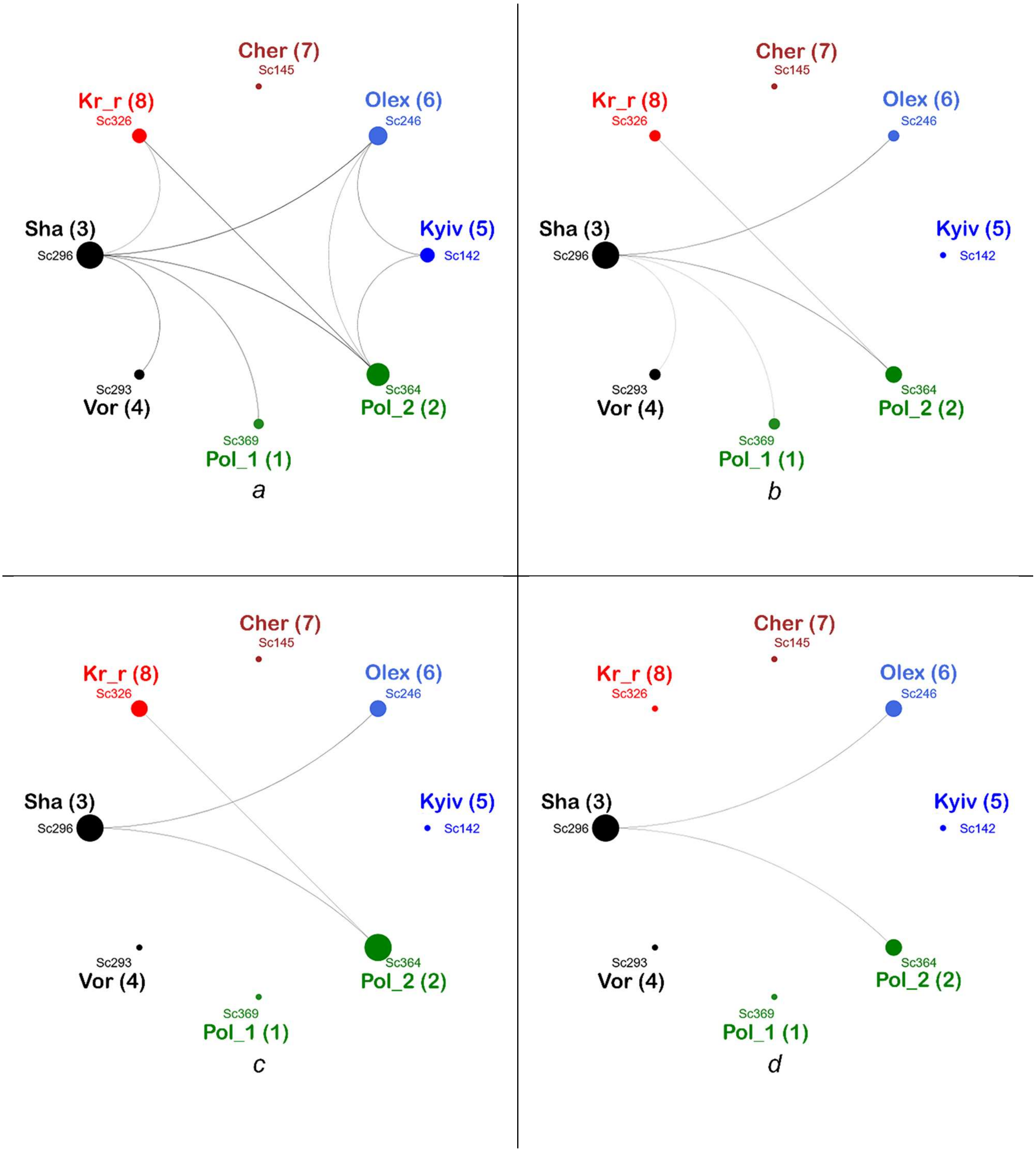

Figure S6 The *S. commune* samples' network of eight locations in Poland and Ukraine at 34% (a), 43% (b), 44% (c), 47% (d) edge cutoff

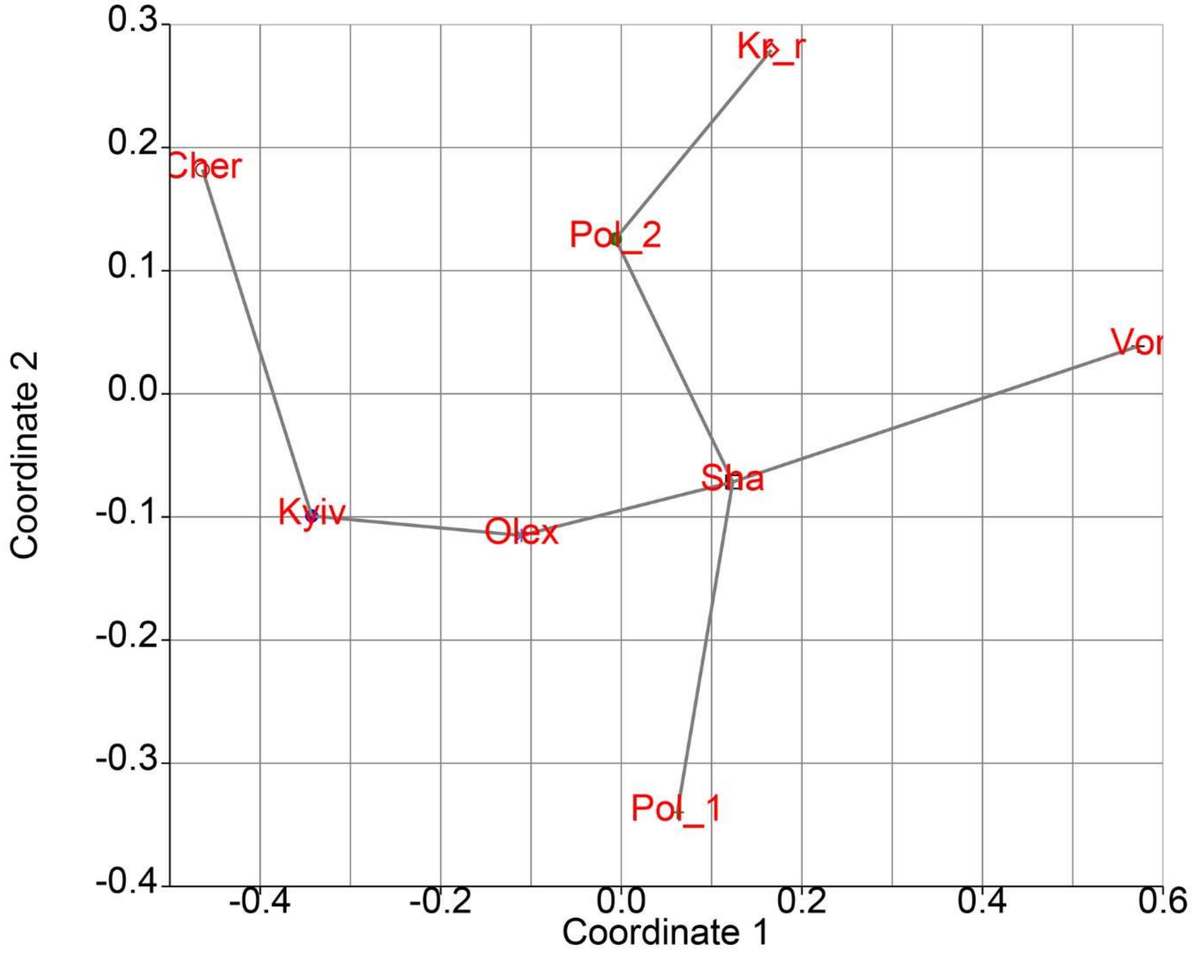

Figure S7 Non-metric multidimensional scaling (Dice similarity index) of the center of genetic alteration samples of *S. commune* (stress: 0.153)

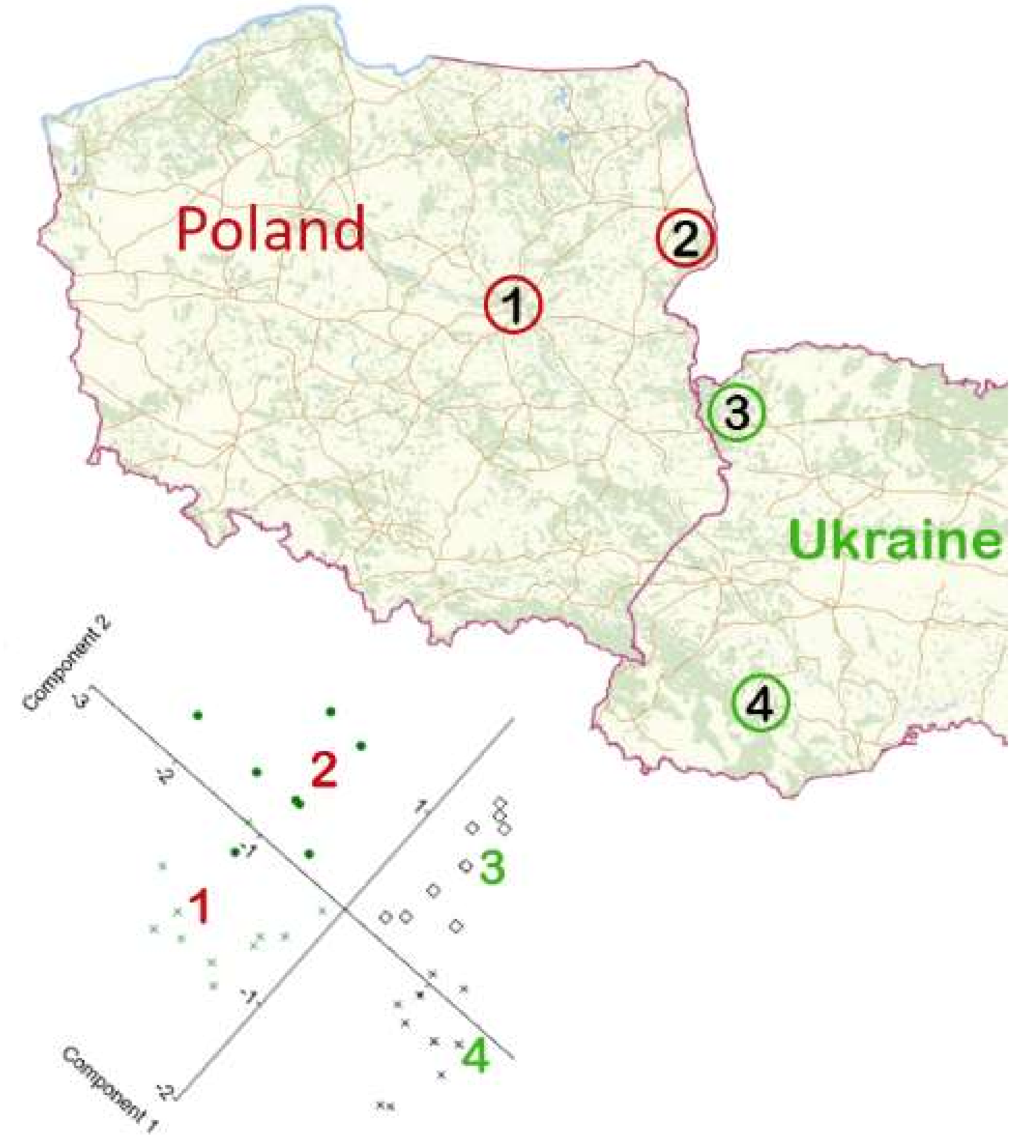

Figure S8 The geographic and principal component space location of subpopulations Pol1 (1), Pol2 (2), Sha (3), and Vor (4) of the *S. commune* fungus
